# Partial ablation of endometrial glands in dogs after exposure to progestin during the neonatal period

**DOI:** 10.21451/1984-3143-2018-0040

**Published:** 2018-08-16

**Authors:** Natália Soares Teixeira, Bianca Barcelos Martins, Rodrigo Volpato, José Leonardo Gualberto Ramos, Patricia Maria Coletto Freitas, Renée Laufer-Amorim, Maria Denise Lopes, Marcelo Rezende Luz

**Affiliations:** 1 Centro de Ciências Agrárias, Universidade Federal do Espírito Santo, Guararema,, Alegre, ES, .; 2 FMVZ, Universidade Estadual Paulista Júlio de Mesquita Filho, Campus de Botucatu, Botucatu, SP, .; 3 Escola de Veterinária, , , .

**Keywords:** ablation, bitch, endometrial glands, receptors, uterus

## Abstract

Bitches with uteri devoid of endometrial glands should be sterile, and consequently could contribute to the population control of dogs. Considering that an inadequate exposure of the female reproductive system to steroids can lead to the formation of the uterine gland knock-out (UGKO) phenotype in some species, the aim of this study was to evaluate the effect of serial applications of medroxyprogesterone acetate (MPA) from birth until the age of six months on the development of endometrial glands in bitches. For this purpose, 16 female mongrel dogs from different litters were distributed into either an MPA group (n = 8), animals treated with 10 mg kg sc (Promone-E^®^, Pfizer, Brasil) at 3-week intervals, from day one after birth until the age of six months, or a control group (n = 8), composed of animals that only received a 0.9% NaCl solution in place of MPA. At six months of age, ovariohysterectomy was performed and uterine horn samples were collected for histological and immunohistochemical examinations. The bitches from the MPA-treated group presented a 35% decrease in the number of endometrial glands, a larger diameter of the endometrial glands, a greater epithelial height, as well as a greater thickness of the uterine wall, endometrium, and myometrium. However, no significant differences were observed between the two groups in the expression of ER-α, ER-β, and PR on the surface epithelium and endometrial stroma. Therefore, the serial application of MPA from birth until the age of 6 months do not completely ablate the development of the endometrial glands in bitches, but impair it by 35%.

## Introduction

Canine overpopulation is a reality in many cities worldwide, particularly in large urban centres, and is a challenge to public health and animal welfare. Dogs act as reservoirs of several diseases, some of which are zoonotic, and pose not only public health risks, but also other problems including traffic accidents ( Maenhoudt *et al*., 2014 ).

Several methods have been used to achieve population control in dogs. The most important include surgical sterilization by ovariohysterectomy (OHE), and hormone therapy. Despite the relative safety and the irreversibility of the sterilization surgery, it is expensive, and can pose risks to the animals ( Howe, 2006 ; Santos *et al*., 2009 ). Moreover, hormone therapies, including the use of progestogens to prevent oestrus, or to block ovulation in female animals, and the use of oestrogens to prevent embryo implantation in fertilized bitches, can have various side effects, including pyometra, breast cancer, and aplastic bone marrow leading to thrombocytopenia and death ( Bowen *et al*., 1985 ; Selman *et al*., 1995 ; Gobello *et al*., 2003 ). New studies have been conducted using analogues of GnRH as a contraceptive agent, and there are also possibilities via the immunological system with vaccination against GnRH, LH receptor and zona pellucida proteins. However, these studies are still in development. Therefore, novel methods of population control are required and may be studied ( Maenhoudt *et al*., 2014 ).

An early event in gestation is attachment of the blastocyst to the uterine wall ( Carter and Mess, 2017 ), which is followed by placentation. The uteri of all mammals contain endometrial glands that synthesize the uterine milk, a complex mixture of substances necessary for the embryo development before placentation ( Perry and Crombie, 1982 ; Gerstenberg and Allan, 1999; Stewart *et al*., 2000 ; Bazer *et al*., 2017 ). Previous studies have demonstrated that the endometrial glands are of fundamental importance for the survival and early development of the foetus ( Bazer, 1975 ; Roberts and Bazer, 1988 ; Carson *et al*., 2000 ; Bazer *et al*., 2017 ).

Steroid hormones have been used to ablate the endometrial glands development in several animal species, including sheep and cows, leading to the formation of the uterine gland knock-out (UGKO) phenotype, characterized by adult female animals rendered infertile by the apparent absence of the endometrial glands and their secretions ( Gray *et al*., 2001 ).

In cattle, new-born calves exposed to progesterone and oestradiol benzoate from birth until puberty, exhibited decreased uterocervical weight and myometrial area, and limited development of endometrial glands ( Bartol *et al*., 1995 ). Similarly, sheep treated with progestins in the neonatal period did not develop endometrial glands ( Bartol *et al*., 1997 ).

In pigs, sheep, and cattle, the development of endometrial glands is a postnatal event, and can occur from birth until the 120^th^ day of life, depending on the species ( Gray *et al*., 2001 ). In dogs, glandular development mainly occurs between birth and the 60th day of life ( Cooke *et al*., 2012 ; Ramos *et al*., 2015 ), however, a single postnatal supraphysiological dose of MPA (35 mg dog sc) was not able to ablate uterine adenogenesis ( Ponchon *et al*., 2015 ).

The UGKO phenotype could lead to sterility in bitches, and consequently, be useful for the development of a population control method. Therefore, the aim of this study was to evaluate the effect of serial applications of medroxyprogesterone acetate (MPA) from birth until the age of six months, on the development of endometrial glands in dogs.

## Materials and Methods

### Animals

Sixteen female mongrel puppies, on day one after birth, were randomly divided into two groups: an MPA group (n = 8), which was treated with doses of MPA (Promone-E^®^, Pfizer, Brasil; 10 mg kg sc; De Bosschere *et al*., 2002 ) at 3-week intervals, from day one after birth until the age of six months, and a control group (n = 8), composed of animals that only received a 0.9% NaCl solution in place of MPA, at the same volume, injected subcutaneously, at the same intervals, until age of six months. The animals were maintained in individual kennels, with access to water and food *ad libitum* , and were clinically evaluated once a week. All the animals were pre-pubertal at the time of ovariohysterectomy, since none of them had presented the first estrus. The weight of the animals at age of six months, when they were ovariohysterectomized, was 10,6 ± 3,8 kg. All the procedures were performed in accordance with the guidelines of the Brazilian College of Animal Experimentation (COBEA).

### Sample collection and histological preparation

Ovariohysterectomy was performed on all the animals at six months of age, and samples of the two uterine horns were collected and fixed for histological examination using haematoxylin-eosin (H & E), and for immunohistochemistry (IHC). A fragment of each uterine horn of each animal was collected, totalling 32 samples.

After fixation in 10% formalin for 24 h, the uterine horn samples were embedded in paraffin. Tissue sections (3-μm thickness) were obtained, mounted on glass slides, and stained with H & E for histological analysis of the type of surface epithelium and for the calculation of the number of endometrial glands. The variables evaluated included the total thickness of the uterine wall (μm), endometrium (μm), and myometrium (μm); diameter of the endometrial glands (μm); number of endometrial glands/mm^2 ^; and the epithelial height (μm). Five fields per histological sample were evaluated for the calculation of the total thickness of the uterine lining, endometrium, and myometrium, and the epithelial height. The glandular diameter was calculated by measuring ten glands per histological sample. The measurements were performed using the ImageJ software version 1.43u (Wayne Rasband, National Institutes of Health, USA).

### Immunohistochemical analysis

Tissue sections (3-μm thickness) were obtained and mounted on glass slides pre-treated with poly-L-lysine (Poly-L-lysine®; Sigma Chemical Co, USA). For the deparaffinization of the embedded material, the slides were maintained in an oven at 55°C for 24 h, and subsequently placed in xylene in a glass beaker, at room temperature for 30 min, and then in fresh xylene for 20 min. The slides were transferred to glass beakers for hydration using alcohol solutions (absolute alcohol solutions I, II, and III, followed by 95 and 85% alcohol solutions), and were treated in each solution for 3 min. Subsequently, the slides were subjected to 10 immersion steps in distilled water. For the detection of ER- α, ER- β and PR, the antigen retrieval was performed in a 10 mM sodium citrate solution with incubation in a Paschal-type pressure cooker (Dako, USA). Endogenous peroxidase was blocked by treatment with 8% hydrogen peroxide for 20 min, after which the slides were washed 5 times in distilled water, blocked with a 3 g/100 ml solution of skimmed milk powder (Molico®) for 60 min, and subjected to 10 immersion steps in distilled water, and 10 washes in Tris buffer pH 7.4 (Trizma Base®, Sigma Chemical Co., USA). The slides were then incubated with the primary antibody (mouse monoclonal anti-human ERα clone 1D5, Cat No. M7047; Dako, CA, USA) at a dilution of 1:50 and a secondary monoclonal antibody (Cat. No. 1546; Immunotech, France) in a moist chamber for 18 h at 4°C. For the detection of ER- β, the primary antibody (mouse monoclonal anti-human ER- β clone PPG5/10, Cat. No. M7292; Dako, CA, USA) was diluted at 1:100, and the slides were incubated in a microwave oven at a maximum power of 750 W for 15 min. For the detection of PR, the primary antibody (monoclonal antibody anti-PR, Cat. No 1546, Immunotech, France), was diluted at 1:50, and the slides were incubated in a moist chamber for 18 h at 4ºC. For all the the receptors, after 18 h of incubation with the primary antibody in a moist chamber at 4°C, the material was then subjected to TRIS bath, and subsequently incubated with the secondary antibody Advance (Advance, Dako, USA) for 30 min each step, even in a moist chamber, according to manufacturer's instructions. Slides were then further washed in TRIS buffer pH 7.4, and revelation with DAB chromogen (3,3'-diaminobenzidine - Liquid DAB Cromogen® - Dako - USA) for 5 min under light and again rinsing with solution TRIS buffer pH 7.4. The slides were counterstained with Mayer's hematoxylin for 30 sec and the staining interrupted with washing in running water for 10 min and five passages in distilled water. The procedure was then dehydrating the material in alcohol baths, when the slides remained for 3 min immersed in each solution (85% alcohol, 95% absolute alcohol I, II, III, xylene I and II) and slides were mounted with coverslips using synthetic resin - Permount (Fisher Scientific - code UN1294e.). Negative controls were treated the same except for replacing the primary antibody with Tris-buffered saline and fractions of rabbit serum immunoglobulins for polyclonal antibody (N-Universal Negative Control Rabbit - DakoCytomation - CA, USA. Code N1699). The expression of the ER- α, ER- β and PR on the surface epithelium and endometrial stroma was evaluated by evaluation and confirmation of positive nuclei staining. A total of five sections were immunostained and evaluated per uterus, with a total of 200 cells with stained nuclei counted in each slide. For analysis, cells from the surface epithelium and endometrial stroma in 10 high power fields (HPF) were selected randomly at magnification 40X for evaluation.

### Statistical analysis

The histological data were evaluated by descriptive analysis, and the morphometric data were analysed by the Tukey’s test using the System for Statistical Analyses (SAS) software version 9.1, 2007. The immunohistochemical data were analysed using the GraphPad Prism 5.0 software (Prism Software, Irvine, CA, USA). Data normality was demonstrated using the Kolmogorov-Smirnoff test. Statistical differences were evaluated using the Student’s t-test, and the analyses were considered significant if the P-value was ≤0.05.

## Results

### Histological analysis

Histological examination indicated that the uteri of the bitches of the MPA group exhibited a simple cylindrical endometrium with a visible lamina propria in the epithelium. The endometrial glands were lined by simple squamous or cylindrical epithelium. In some cases, the myometrium presented large, conspicuous, irregular blood vessels. In the bitches of the control group, the uterine endometrium exhibited simple cuboidal epithelium, endometrial glands, and a tubular lamina propria with cubic and/or cylindrical epithelium with the latter type showing irregular folds. Smooth muscle layers with large vessels were also observed in the myometrium.

The bitches from the MPA-treated group presented a significant decrease in the number of the endometrial glands (P ≤ 0.05) compared to the control group, a larger diameter of the endometrial glands (P ≤ 0.05), a greater epithelial height (P ≤ 0.05), as well as a greater thickness of the uterine wall (P ≤ 0.05; Table 1 ).

**Table 1:** Mean (± S.E.M.) number of endometrial glands/mm^2^, glandular diameter (µm), thickness of the uterine wall (µm), endometrium (µm), myometrium (µm) and epithelial height (µm) from bitches treated with MPA from birth to 6 months of age, or control (C).

Structure	MPA	C
Endometrial glands/mm^2^	6.83 ± 0.43^a^	9.51 ± 0.39^b^
Glandular diameter	42.23 ± 1.36 ^a^	30.97 ± 0.93^b^
Thickness of the uterine wall	1526.33 ± 21.06^a^	1326.75 ± 50.56^b^
Endometrium	613.67 ± 10.45^a^	463.40 ± 32.06^b^
Myometrium	923.26 ± 12.50^a^	870.77 ± 14.32^b^
Epithelial height	9.04 ± 0.06^a^	7.44 ± 0.13^b^

Means followed by different letters at the same line differed statistically by Tukey´ *s* test (P ≤ 0.05).

### Immunohistochemistry

**Figure1:**
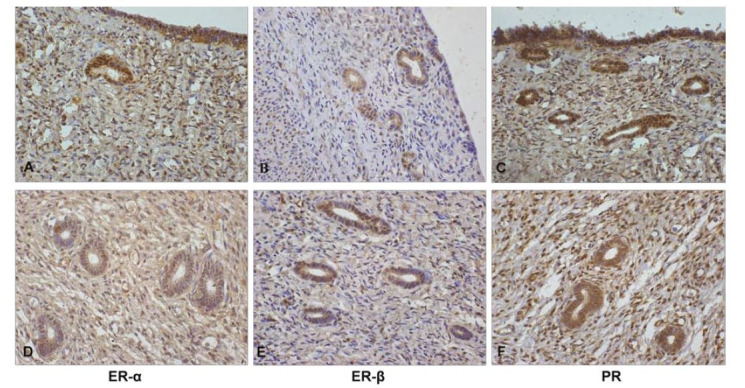
Photomicrographs (X 40) of immunohistochemical sections of surface epithelium and endometrial stroma from bitches treated or not treated with MPA from birth to 6 months of age. (A and D) expression of ER-α, (B and E) expression of ER-β, and (C and F) expression of PR on the surface epithelium and endometrial stroma, respectively.

There was no statistical difference compared to ER- α expression (P = 0.8330), ER- β (P = 0.7209) and PR (P = 0.3181) in the superficial epithelium, between the MPA and C groups, respectively. Likewise, in the endometrial stroma no statistical difference was observed in ER- α expression (P = 0.5990), ER- β (P = 0.1949) and PR (P = 0.1873), respectively ( Table 2 ; Fig. 1 ).

**Table 2:** Mean (± S.E.M.) of stained nuclei for ER- α, ER- β and PR on the surface epithelium and endometrial stroma, from bitches treated with MPA from birth to 6 months of age, or control (C).

	MPA	C
Surface epithelium		
ER-α	88.50 ± 10.04^a^	87.75 ± 9.662^a^
ER-β	23.25 ± 36.16^a^	26.75 ± 23.18^a^
PR	68.25 ± 15.65^a^	81.25 ± 22.57^a^
		
Endometrial stroma		
ER-α	66.75 ± 8.799^a^	73.75 ± 26.27^a^
ER-β	49.00 ± 23.99^a^	24.00 ± 13.47^a^
PR	74.50 ± 8.932^a^	66.25 ± 15.90^a^

Means followed by the same letter at the same line do not differ statistically by Student´s T test.

### Clinical manifestations

All the animals in the MPA group presented with a mild breast hypertrophy, which began shortly after the first treatment. This hypertrophy decreased after the last application of MPA at 6-months of age, but did not completely disappear. The treated animals also presented with clitoral hypertrophy starting at 4-months of age, with a complete regression at 19-months of age. No side effects were observed in the control group. The animals were monitored until the age of 24 months.

## Discussion

In this study, serial injections of MPA in dogs, from birth until the age of six months, did not completely ablate the endometrial glands, but decreased their development by approximately 35%. Similarly, a total or partial ablation of the endometrial glands was observed in cows that received progesterone in combination with oestradiol benzoate ( Bartol *et al*., 1995 , 1997 ). However, neonatal dogs which received only a single postnatal supraphysiological dose of MPA (35 mg dog sc) presented a normal uterine adenogenesis ( Ponchon *et al*., 2015 ). It is known that an inadequate exposure of the female reproductive tract to steroids during development can disrupt the physiological events necessary for growth and differentiation, leading to altered phenotypes in adults, and consequently, lower reproductive efficiency ( Gray *et al*., 2001 ). Furthermore, cows treated with progesterone in combination with oestradiol benzoate in the neonatal period had lower pregnancy rates because of the underdevelopment of the endometrial glands ( Bartol *et al*., 1995 ). This might have been due to the decreased synthesis or transport of essential substances (uterine milk) that are essential for foetal survival and development during the peri-implantation period ( Gray *et al*., 2001 ). The differences between our results and those from Ponchon *et al*. (2015) may be due to the frequency of MPA used, and not dosage, since the period of exposure to hormones is directly associated with distinct effects on organ development and reproductive functions ( Cooper and Kavlock, 1997 ).

The diameter of the endometrial glands was larger in the bitches of the MPA group, compared with those of the control group. This increase in diameter was due to the prolonged action of MPA, which is a progestogen with progesterone-like effects. It increases the glandular secretory capacity, which justifies the larger glandular diameter found in dogs of the treated group. The physiological effects of progesterone are mediated by the interaction of the hormone with its receptor (PR), which was normally expressed on the surface epithelium and endometrial stroma of the treated dogs in the present study ( Muulac-Jericevic and Conneely, 2004 ).

The thickness of the uterine wall, endometrium, and myometrium, and the height of the surface epithelium were greater in the bitches treated with MPA by the time of ovariohysterectomy, compared to those in the control. The increased thickness of the endometrium and the uterine wall may be due to the glandular dilation and secretion, as described in adult dogs treated by the same protocol ( De Bosschere *et al*., 2002 ). All these changes might be caused due to stimulation by MPA since, in cyclic bitches, luteal progesterone also causes an increase in the thickness of the uterine wall ( Romagnoli and Concannon, 2003 ).

In this study, ER α, ER β, and PR were normally expressed on the surface epithelium and endometrial stroma ( Fig. 1 ), without any statistical difference in their expression between the two groups. These results are in contrast with others, in which lower expression levels of the ER, and none of the PR, in the glandular epithelium were observed, but a moderate or high expression of the PR in other regions of the endometrium of dogs, after treatment with progesterone or MPA was detected ( Dhaliwal *et al*., 1999 ). In fact, progesterone decreases the number of oestrogen receptors (ERs) and PRs in the uterine tissues of some animals, including bitches and cats, *in vivo* ( West *et al*., 1976 , 1977 ; Vermeirsch *et al*., 1999 , 2000 ), and even *in vitro* ( Galabova *et al*., 2004 ). However, it has been described normal expression of the ER and PR in adult bitches treated for 24 weeks by the same protocol used in the present study ( De Bosschere *et al*., 2002 ), indicating, as observed in our study, that the sex-hormone receptors in most cell types of the uterine wall escape the down-regulation promoted by the progestins after prolonged exogenous administration. In addition, it is known that the expression of the ER and PR may occur regardless of the regulation by sex hormones ( Okulicz, 1986 ; Clarke *et al*., 1990 ).

Our result for the expression of PR in the bitches of the control group differed from another study which could not detect the expression of the PR in the reproductive system of immature Beagle bitches not subjected to hormone treatment ( Lessey and Gorell, 1981 ). However, our result is in agreement with a recent description of the expression of both the ER and PR in the uterus of one-week-old puppies ( Cooke *et al*., 2012 ).

In this study, the only clinical manifestation observed was a mild enlargement of the breasts and the clitoris. This outcome was due to the presence of progesterone receptors in the mammary gland ( Concannon *et al*., 1981 ), and probably due to the androgenic effects of progestogens on the clitoris ( Romagnoli and Concannon, 2003 )

The development of a knockout canine experimental model would expand the knowledge on implantation, placentation, and normal embryonic development, as well as the study of the development of protocols for birth control in the species. However, it was not possible to produce a UGKO model in this study, with the protocol used. Similarly, oestrogen and progesterone administration from birth until the age of six months, was not capable to ablate the endometrial glands on fillies ( Wilsher *et al*., 2009 ).

In conclusion,the use of exogenous progestin is capable of causing partial ablation of the development of the endometrial glands in dogs, with normal expression of ER α and β and PR.

Conflict of interest

## References

[B001] Bartol FF, Johnson LL, Floyd JG, Wiley AA, Spencer TE, Buxton DF, Coleman DA (1995). Neonatal exposure to progesterone and estradiol alters uterine morphology and luminal
protein content in adult beef heifers. *Theriogenology*.

[B002] Bartol FF, Wiley AA, Spencer TE, Ing NH, Ott TL, Bazer FW (1997). Progestin exposure from birth: epigenetic induction of a unique adult uterine phenotype
in sheep a glandless endometrium. *Biol Reprod*.

[B003] Bazer FW (1975). Uterine protein secretions: relationship to development of the conceptus. *J Anim Sci*.

[B004] Bazer FW, Wu G, Johnson GA (2017). Pregnancy recognition in mammals: the roles of interferons and estrogens. *Anim Reprod*.

[B005] Bowen RA, Olson PN, Behrendt MD, Wheeler SL, Husted PW, Nett TM (1985). Efficacy and toxicity of estrogens commonly used to terminate canine pregnancy. *J Am Vet Med Assoc*.

[B006] Carson DD, Bagghi I, Dey SK, Enders AC, Fazleabas AT, Lessey BA, Yoshinaga K (2000). Embryo implantation. *Dev Biol*.

[B007] Carter AM, Mess AM (2017). The evolution of fetal membranes and placentation in carnivores and ungulates (Ferungulata). *Anim Reprod*.

[B008] Clarke IJ, Horton RJE, Doughton BW (1990). Investigation of the mechanism by which insulin-induced hypoglycemia decreases luteinizing
hormone secretion in ovariectomized ewes. *Endocrinology*.

[B009] Concannon PW, Spraker TR, Casey HW, Hansel W (1981). Gross and histopathologic effects of medroxyprogesterone acetate and progesterone on
the mammary glands of adult beagle bitches. *Fertil Steril*.

[B010] Cooke PS, Borsdorf DC, Ekman GC, Doty KF, Clark SG, Dziuk PJ, Bartol FF (2012). Uterine gland development begins postnatally and is accompanied by estrogen and progesterone
receptor expression in the dog. *Theriogenology*.

[B011] Cooper RL, Kavlock RJ (1997). Endocrine disruptors and reproductive development: a weight-of-evidence overview. *J Endocrinol*.

[B012] De Bosschere H, Ducatelle R, Tshamala M, Coryn M. (2002). Changes in sex hormone receptors during administration of progesterone to prevent estrus
in the bitch. *Theriogenology*.

[B013] Dhaliwal GK, England GC, Noakes De W (1999). The influence of exogenous steroid hormones on steroid receptors, uterine histological
structure and the bacterial flora of the normal bitch. *Anim Reprod Sci*.

[B014] Galabova GK, Walter I, Aurich C, Aurich JE (2004). Steroid receptors in canine endometrial cells can be regulated by estrogen and progesterone
under *in vitro* conditions. *Theriogenology*.

[B015] Gerstenberg C, Allen WR (1999). Development of the equine endometrial glands from fetal life to ovarian cycling. *J Reprod Fertil*.

[B016] Gobello C, Castex G, Klima L, Rodríguez R, Corrada Y. (2003). Study of two protocol combining aglepristone and cloprostenol to treat open cervix pyometra
in the bitch. *Theriogenology*.

[B017] Gray CA, Bartol FF, Tarleton BJ, Wiley AA, Johnson GA, Bazer FW, Spencer TE (2001). Developmental biology of uterine glands. *Biol Reprod*.

[B018] Howe LM (2006). Surgical methods of contraception and sterilization. *Theriogenology*.

[B019] Lessey BA, Gorell TA (1981). Nuclear progesterone receptors in the beagle uterus. *J Steroid Biochem*.

[B020] Maenhoudt C, Santos NR, Fontbonne A (2014). Supression of fertility in adult dogs. *Reprod Domest Anim*.

[B021] Muulac-Jericevic B, Conneely OM (2004). Reproductive tissue selective actions of progesterone receptors. *Reproduction*.

[B022] Okulicz WC (1986). Progesterone receptor replenishment during sustained progesterone treatment in the
hamster uterus. *Endocrinology*.

[B023] Perry JS, Crombie PR (1982). Ultrastructure of the uterine glands of the pig. *J Anat*.

[B024] Ponchon T, Merlo ML, Faya M, Priotto M, Barbeito C, Gobello C (2015). Postnatal exposure to a progestin does not prevent uterine adenogenesis in domestic dogs. *J Vet Sci*.

[B025] Ramos JLG, Ramos CLFG, Cunha ICN, Carvalho ECQ, Shimoda E, Luz MR (2015). Análise histomorfométrica do útero na espécie canina
do nascimento aos seis meses de idade. *Arq Bras Med Vet Zootec*.

[B026] Roberts RM, Bazer FW (1988). The function of uterine secretions. *J Reprod Fertil*.

[B027] Romagnoli S, Concannon PW, Concannon PW, England G, Verstegen J, Linde-Forsberg C (2003). Clinical use of progestins in bitches and queens: a review.. Recent Advances in Small Animal Reproduction..

[B028] Santos FC, Corrêa TP, Rahal SC, Crespilho AM, Lopes MD, Mamprim MJ (2009). Complicações da esterilização cirúrgica de fêmeas
caninas e felinas. Revisão de literatura. *Vet Zootec*.

[B029] Selman PJ, Van Garderen E, Mool JA, Van Den Ingh TS (1995). Comparison of the histological changes in the dog after treatment with the progestins medroxyprogesterone
acetate and proligestone. *Vet Q*.

[B030] Stewart T, Sage R, Stewart A, Cameron D. (2000). Breast cancer incidence highest in the range of one species of house mouse, *Mus
domesticus.*. *Br J Cancer*.

[B031] Volpato R. (2011). Avaliação clínica e imunoistoquímica do útero
e cérvix de cadelas com o diagnóstico de piometra.

[B032] Vermeirsch H, Simoens P, Hellemans A, Coryn M, Lauwers H. (2000). Immunohistochemical detection of progesterone receptors in the canine uterus and their
relation to sex steroid hormone levels. *Theriogenology*.

[B033] Vermeirsch H, Simoens P, Lauwers H, Coryn M. (1999). Immunohistochemical detection of estrogen receptors in the canine uterus and their relation
to sex steroid hormone levels. *Theriogenology*.

[B034] West NB, Verhage HG, Brenner RM (1976). Suppression of the estradiol receptor system by progesterone in the oviduct and uterus
of the cat. *Endocrinology*.

[B035] West NB, Verhage HG, Brenner RM (1977). Changes in nuclear estradiol receptor and cell structure during estrous cycles and pregnancy
in the oviduct and uterus of cats. *Biol Reprod*.

[B036] Wilsher S, Lefranc AC, Allen WR (2009). Post natal oestrogen administration stimulates precocious endometrial gland development
in the horse. *Equine Vet J*.

